# Long-Term Outcome of Thrombolytic Therapy for Massive Pulmonary Embolism in Pregnancy

**DOI:** 10.1155/2020/9153618

**Published:** 2020-02-19

**Authors:** Sy Van Hoang, Anh Tuan Vo, Kha Minh Nguyen

**Affiliations:** ^1^Department of Internal Medicine Faculty of Medicine, University of Medicine and Pharmacy at Ho Chi Minh City, Vietnam; ^2^Department of Cardiovascular Surgery, University Medical Center, University of Medicine and Pharmacy at Ho Chi Minh City, Vietnam

## Abstract

A 25-year-old pregnant woman presented at 12 weeks of gestation with syncope and shortness of breath caused by massive pulmonary embolism. Due to persistent shock, fibrinolytic therapy with rtPA was administered. After fibrinolysis, clinical and hemodynamic response was excellent. No bleeding and fetal complications were recorded.

## 1. Introduction

Pulmonary embolism in pregnancy is still a leading cause of direct maternal mortality in the world [1]. During pregnancy, three pathophysiological mechanisms such as venous stasis, vascular injury, and hypercoagulability, known as Virchow's triad, that are together or in isolation may be responsible for the high incidence of pulmonary embolism in pregnancy [2]. Diagnosing of PE in pregnancy faces a number of difficulties due to clinical symptom overlaps due to hemodynamic changes during pregnancy period, as well as limitations of clinical indication (imaging) because it can affect the fetus [3].

In clinical trials, especially interventional trials, pregnant women are potentially vulnerable, and the fetus is faced with danger. Almost all clinical trials were done using methods regarding the diagnostic strategies and treatment of pulmonary embolism that exclude pregnant women due to moral and ethical standards by critics [4]. The recent guidelines for the management of cardiovascular diseases during pregnancy were developed by extrapolating the evidence for nonpregnant patients and had the weak-quality evidence [5]. A massive pulmonary embolism is an extremely life-threatening situation for the mother and fetus. The management of massive pulmonary embolism in nonpregnant patients is based on the acute removal of the thrombotic clot in the pulmonary arteries such as systemic thrombolysis, percutaneous catheter-based embolectomy, or surgical embolectomy. But clinicians face the challenge of treating pregnant patients with massive pulmonary embolism because of a lack of high-quality evidence to inform and guide their treatment decisions. Therefore, available data from case reports, case series, or retrospective observational studies are collected that is extremely helpful in treating massive pulmonary embolism in pregnant patients. In order to fill this important literature gap, we demonstrate a case report of massive pulmonary embolism in pregnancy treated by systemic thrombolysis (alteplase).

## 2. Case Report

A 25-year-old pregnant woman at 12 weeks of gestation was brought to the emergency department of our hospital by ambulance with worsening shortness of breath on exertion and onset of sudden syncope. The patient denied any cardiac disease and using any medication and had no relevant family history. She was having a swollen right leg from the ankle to the knee and dyspnea on exertion for the past week.

On admission, the patient was agitated and tired, and there is evidence of decompensation condition such as hypotension (70/40 mmHg), tachycardia (132 bbp), tachypnea (40 cpm), and low oxygen saturation in oxygen with a nonrebreather mask (8 L/min). She had signs of deep venous thrombosis in the unilateral leg. Jugular vein distention was visible, her lung fields were clear, and findings from cardiac examination were tachycardia, no gallop S3, S4, and existence of a 2/4 high-pitched systolic murmur heard along the upper left sternal border.

**Figure 1:**
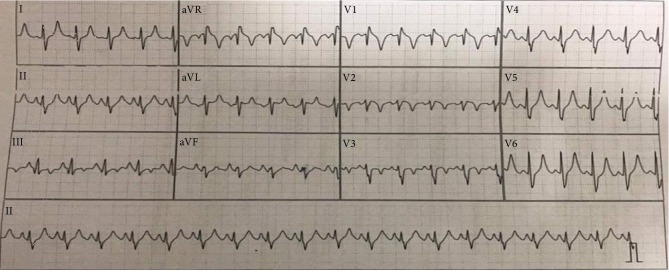
12-lead ECG showing sinus tachycardia with a typical S1-Q3-T3 pattern.

**Figure 2:**
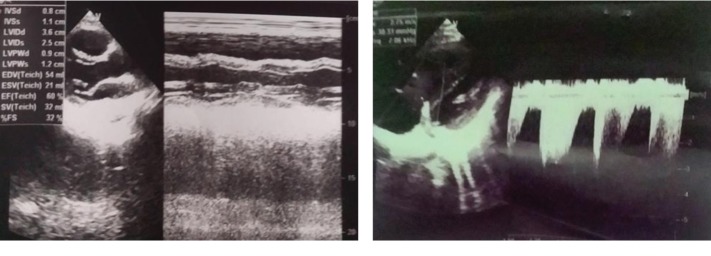
The transthoracic echocardiography on admission showing the right ventricular dilation, severity of tricuspid regurgitation, moderate pulmonary arterial hypertension with PAPs 50 mmHg, and normal left ventricular ejection fraction.

Laboratory tests revealed hemoglobin 132 g/L, WBC 12 G/L, PLT 155 G/L, D-Dimer >10,000 ng/mL, and Troponin I 1.482 ng/mL. The electrocardiogram showed sinus tachycardia and signs of right ventricular overload with a typical S1-Q3-T3 pattern (Figure 1). The transthoracic echocardiography performed on admission revealed signs of the right ventricular failure such as the right ventricular dilation, severity of tricuspid regurgitation, moderate pulmonary arterial hypertension with PAPs 50 mmHg, and normal left ventricular ejection fraction (Figure 2). Additional, deep venous thrombosis in the right leg was confirmed by duplex ultrasound, and obstetric ultrasound showed a fetus with cardiac pulsations inside the uterus at 12 weeks.

**Figure 3:**
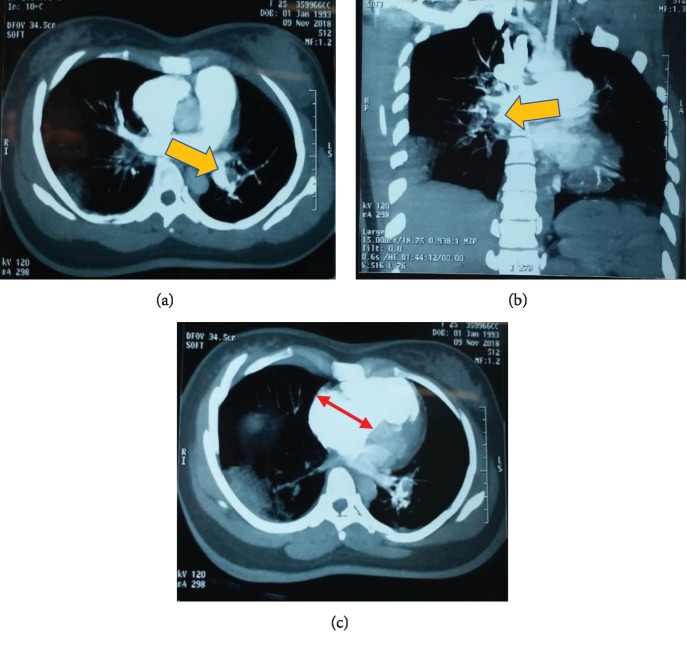
Thoracic computed tomography (CT) with intravenous contrast. Bilateral pulmonary thromboembolism (yellow arrow) in (a) coronal plane and (b) frontal plane; (c) shows the right ventricular dilation with the left ventricular compression (red 2-headed arrow).

According to some evidence of clinical signs, laboratory tests, and initial imaging, a diagnosis of massive pulmonary embolism is suspected. In a patient with suspected massive pulmonary embolism who is in critical condition such as in an extremely life-threatening situation for the mother and the fetus, it was necessary to perform thoracic computed tomography (CT) with administration of intravenous contrast in the pulmonary arterial phase, with the fetus being protected from radiation by lead shielding. CT angiography revealed the right ventricular dilation (Figure 3(c)) and acute bilateral pulmonary thromboembolism (Figures 3(a) and 3(b)).

After first aid consisting of intravenous line placement, oxygen supplementation, subcutaneous enoxaparin 1 mg/kg, fluid infusion, and a vasoconstrictor agent (noradrenaline with dose of 0.5 mcg/kg/min), she was transferred to the CCU (coronary care unit) with a diagnosis of massive pulmonary embolism. Although there was a relative contraindication to thrombolysis, the condition of the mother and the fetus was extremely dangerous; systemic thrombolytic therapy was administered with alteplase (10 mg bolus, then 90 mg over 2 h) and according to partial thromboplastin time for the first 48 h, when enoxaparin 1 mg/kg twice daily was started. Within 2 hours, remarkable clinical and hemodynamic improvement was seen, with blood pressure 90/65 mmHg, heart rate 98 bpm, breathing rate 25 cpm, and decreasing need for oxygen supplementation, and dosage of noradrenaline was titrated based on the patient's response. After 6 hours of termination of fibrinolytic therapy, hemodynamic variables were stable, and she was weaned off noradrenaline infusion. She was discharged from the CCU on the third day.

**Figure 4:**
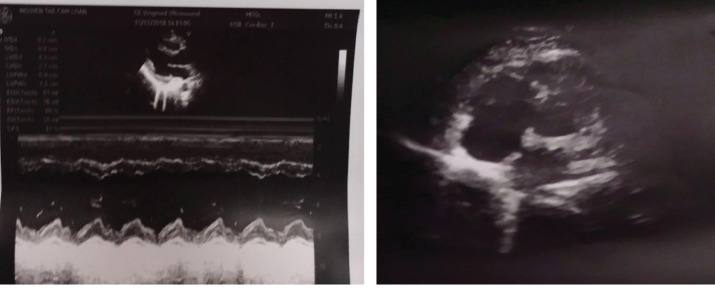
The transthoracic echocardiography on the fourth day showing the normal right ventricular dimension and the normal left ventricular ejection fraction.

On the fourth day, transthoracic echocardiogram (Figure 4) and obstetric ultrasound were performed, which revealed the normal right ventricular dimension, no signs of pulmonary hypertension, and no abnormal signs of pregnancy. She was discharged home on the 12th day at 14 weeks, medicated with acenocoumarol. Acenocoumarol was switched to subcutaneous enoxaparin from the 24th week of pregnancy to delivery. The patient was followed up regularly by experts in cardiology and obstetrics. On the 30th day after discharge, obstetric ultrasound revealed fetal growth in the same percentile curve and normal morphology with biparietal diameter 42 mm and the length of the femur 26 mm and amniotic fluid volume. At 36th week, the patient gave birth to a healthy 2.6-kilo baby boy. The anticoagulation was discontinued at 2 months postpartum.

## 3. Discussion

### 3.1. Diagnosis

It is usually difficult to evaluate the clinical signs of thromboembolism in pregnancy because most of them have bilateral leg edema, and there are 70% of pregnant women having dyspnea as well [6]. According to risk factor and clinical signs, it is reasonable to estimate the risk of pulmonary embolism in patients by using Wells score or Geneva score. These scores, however, are not used for pregnancy. In this case, the patient only had both leg edemas before presenting more severe symptoms and signs.

The clinical signs, laboratory tests, and initial imaging suggested the diagnosis of massive pulmonary embolism that could impair hemodynamics in this patient. The problem was that the fetus had to be exposed to radiation when performing thoracic CT with administration of intravenous contrast as a diagnostic test. The results would provide more information for the optimal and suitable treatment strategy to save the patient. Some studies showed that a fetus was exposed to the radiation of <0.01 mSv and 0.24–0.66 mSv with each chest X-ray and thoracic CT, respectively. However, the fetal vulnerability threshold is 50 mSv [7]. These necessary diagnostic imaging techniques have little effect on the fetus and conduct more benefits than risks for patients and fetuses [2]. In this situation, we requested a thoracic CT scan with pulmonary angiography at the recommendation level of IIA according to ESC 2014 guidelines [8].

### 3.2. Treatment

Regarding the treatment, this patient was diagnosed as having massive pulmonary embolism impairing hemodynamics at 12-week gestation. Pregnancy is a relative contraindication to thrombolysis [8]. The current treatment guidelines recommend that thrombolytic therapy is considered in pregnant patients whose life is at stake as this patient. Up to now, no comparison of the efficacy between thrombolytic agents in pregnancy has been reported, but the current treatment guidelines recommend using rtPA or tenecteplase rather than urokinase/streptokinase because of their safety and efficiency [9]. If patients can be indicated to deliver (fetus can be alive), thrombolytic therapies should not be used except for the extremely life-threatening conditions or the case when thrombectomy and delivery cannot be carried out. The doctors have to consider the benefits and the risks when using thrombolytic therapies. The hemodynamic variables became gradually stable after usage of rtPA. In addition, the fetus was still alive and did not reveal any abnormalities according to obstetric ultrasound. Similarly, Ahearn et al. reported a case that a 12-week gestational 36-year-old woman who required massive pulmonary embolism with the shock condition was treated successfully with rtPA (dose of 100 mg IV in 2 hours) [9]. In 2019, a report about the outcome of thrombolytic therapies in 141 severe thromboembolic pregnant patients shows that there are 4 deaths (2.8%), 12 cases of severe hemorrhage (8.5%), 13 cases of mild/intermediate hemorrhage (9.2%), 9 cases of miscarriage (6.4%), 2 cases of stillbirth (1.4%), and 14 cases of premature delivery (9.9%) [10]. In our case, she gave birth to a healthy 2.6-kilo baby boy at 36th week. So this case was among the cases of premature delivery. At 36 weeks, this baby is considered late preterm then the risk of premature complications decreases significantly.

The method that thrombolytic agents are given locally via a catheter requires available instruments and experienced specialists, and there is no evidence that this method is more efficient than systemic thrombolytic therapy. Thrombectomy of pulmonary arterial embolism in impaired hemodynamic pregnancy has been reported in the world. However, the recommendation of surgery and criteria for patient inclusion are still controversial [11]. Many authors reported some cases of successful thrombectomy in pregnant women with massive pulmonary embolism. Blegvad et al. reported 10 cases of emergent thrombectomy for massive pulmonary embolism during pregnancy. Accordingly, there was no case of maternal death but 4 cases of miscarriage [12].

## 4. Conclusion

Pulmonary thromboembolism is common in pregnancy, and clinicians should keep a diagnosis of pulmonary embolism in mind, especially in the presence of suspicious clinical signs, laboratory tests, and initial imaging, including electrocardiogram and echocardiogram. Massive pulmonary embolism is associated with significant maternal and fetal mortality. Detecting early diagnosis and reasonable treatment strategies for massive pulmonary embolism improve the long-term outcome for the pregnant woman and fetus. Current evidence showed that the benefits of imaging tests outweigh the potential radiation risks when using radioactive sources such as thoracic computed tomography to diagnose pulmonary embolism. The thrombolytic therapy should be considered for life-threatening pulmonary embolism during pregnancy.
